# Dental and periodontal dimensions stability after esthetic clinical crown lengthening surgery: a 12‐month clinical study

**DOI:** 10.1007/s00784-023-05458-5

**Published:** 2024-01-05

**Authors:** Valéria Martins de Araújo Carneiro, Andressa Meireles Seabra Gomes, Mônica Umpierre Marinho, Gabriel Simino de Melo, Feras Kasabji, Tien-Li An, Cristine Miron Stefani, Maria do Carmo Machado Guimarães, Carlos Alexandre Soares Andrade

**Affiliations:** 1 Faculdade de Ciências da Saúde, Departamento de Odontologia, Universidade de Brasília, Campus Universitário Darcy Ribeiro, Asa Norte, Brasília-DF, 70910-900 Brazil; 2 Faculdade de Medicina e Odontologia, Departamento de Periodontia, Faculdade São Leopoldo Mandic, Rua Dr. José Rocha Junqueira 13, Campinas-SP, 13045-755 Brazil; 3 Faculty of Medicine, Department of Public Health and Epidemiology, University of Debrecen, Kassai Út 26, 4028 Debrecen, Hajdú-Bihar, Hungary

**Keywords:** Crown lengthening, Periodontium, Gingiva, Alveolar process, Dental esthetics

## Abstract

**Objectives:**

To evaluate the stability of periodontal tissues 3 (T_3_), 6 (T_6_), and 12 (T_12_) months after esthetic crown lengthening (ACL) and the possible correlations between changes in those structures.

**Materials and methods:**

Twenty individuals were evaluated through clinical assessment, photography, and tomography. Measurements included gingival margin (GM), clinical crown length (CCL), interdental papilla height (PH) and width (PW), gingival thickness (GT), bone thickness (BT), probing depth (PD), distance between alveolar crest and GM, distance between alveolar crest and cementoenamel junction. Nonparametric and correlation statistics were performed (*p* < 0.05).

**Results:**

CCL at T_0_ was 7.42 ± 0.70 mm and increased to 9.48 ± 0.49 mm immediately after ACL, but it decreased to 8.93 ± 0.65 mm at T_12_. PD decreased 0.60 mm from T_0_ to T_6_, and it increased 0.39 mm from T_6_ to T_12_. BT decreased 0.20 mm, while GT increased 0.29 mm from T_0_ to T_12_. Both PW and PH showed enlargement in T_12_. A positive moderate correlation was found between CCL/T_0_ and CCL/T_12_, GT/T_0_ and AC-GM/T_12_, BT/T_0_ and GT/T_12_. A few negative moderate correlations were PD/T_0_ and CCL/T_12_, PD/T_0_ and PH/T_0_, PD/T_0_ and BT/T_12_.

**Conclusions:**

ACL procedure was effective. Although some rebound occurred, that was not clinically important. PD tended to reestablish its original length, partially due to a migration of GM during the healing period. Besides, a thickening of supracrestal soft tissues was observed.

**Clinical relevance:**

The present study centers on the factors influencing the stability of periodontal tissues after esthetic crown lengthening, underscoring the procedure’s influence on esthetics and biology and the need for careful treatment planning.

## Introduction

Dentistry is currently facing massive changes regarding the high esthetic demand from patients [1]. The pursuit for an esthetic smile comprises not only the color, size, or shape of the teeth but also periodontal tissues characteristics [2, 3]. Excessive gingival display (EGD), commonly known as “Gummy smile”, is a multifactorial non-pathological condition found in 10.5–29% of the population, being more prevalent in young and female individuals [4–6]. EGD is a condition associated with dental, periodontal, and musculoskeletal factors. The most common associated factor is a periodontal condition called altered passive eruption (APE), responsible for approximately 12.1 to 35.8% of the diagnosed cases of EGD [7, 8]. This condition can be found in adult individuals in which the gingival margin (GM) failed to migrate into the cementoenamel junction (CEJ) direction causing short clinical crowns where a large amount of enamel is covered by gingival tissue [9, 10]. Despite being a non-pathological periodontal condition, some evidence suggests that individuals with APE have a higher risk of developing periodontal diseases [8, 11, 12]. The most suitable treatment for EGD, caused by APE, is the esthetic crown lengthening procedure (ACL). It consists of a periodontal resective surgery, associated with osteotomy and osteoplasty in most cases [7, 13].

Pre-restorative crown lengthening surgery (CL) has been widely assessed in several studies across the decades [14–16]. Factors regarding the stability of GM outcomes might comprise variables such as clinician skills, periodontal phenotype, surgical technique, distance of the flap, and tooth type. A rebound of the condition could be more related to molars, thick phenotype, inter-individual variability of biologic width, and less than 3 mm of the distance between alveolar crest (AC) and GM left at the time of suture [14–18]. Evidence demonstrates that—following CL procedure—a new supracrestal gingival complex might be presented with modified dimensions such as 0.45 mm shorter compared to the same structure before surgery [15]. In the same study, the authors state that greater bone reduction is required, particularly in individuals presenting thick phenotype. The amount of rebound can also be caused by technical aspects or individual variations during the healing process, not being influenced by gender or age [14, 18, 19].

Despite the amount of evidence regarding the pre-restorative crown lengthening procedure, it is important to acknowledge the difference between this procedure and ACL to treat APE [20]. The first difference within the procedures is anatomical, since ACL is mostly performed in buccal sites of maxillary anterior teeth (or esthetic region) [13], and CL can be done in any site of any teeth depending on the restorative needs [16]. Moreover, CL usually comprises interproximal sites, which is not indicated in ACL, since the esthetic outcomes of the interdental papillae are critical [21]. Another difference regards the histological characteristic of APE periodontal tissues in comparison to regular tissue since the supracrestal tissues are genetically elongated, more prone to gingival inflammation, and present exacerbated inflammatory response in epithelial level [7, 8, 11]. Therefore, tissue rebound in patients with APE, who underwent ACL, should be assessed in a different perspective, since the healing and maturation of gingival tissues are distinctive from usual cases [12].

Furthermore, the progression of probing depth (PD) and dimensional changes in interdental papillae after ACL are still neglected topics in the literature, albeit their relevance. The sulcular epithelium average measures 0.69 mm (range 0.61 – 1.71 mm), which should be differentiated from the clinical gingival sulcus probing depth that ranges from 1 to 3 mm in a healthy site, considering that the periodontal probe clinically penetrates the coronal part of the junctional epithelium (average 0.97 mm, 0.71 – 1.35 mm) [22, 23]. Evidence suggests that the PD presents a statistically significant decrease in central incisors (CI), lateral incisors (LI), and canines (CN) from baseline to a short follow-up of 6 months after ACL [20] or CL [19].

The baseline dimensions of the papilla is one of the diagnostic criteria to be considered in APE since those patients present the papilla base width disproportionate compared to the height [7, 24]. The most appropriate methods to preserve papilla height and minimize interdental soft tissue rebound are resecting only the buccal sites of the papilla, considering an interdental split flap, and avoiding interdental bone removal [25]. However, how the dimensional modification of the interdental papilla occurs after ACL is still a lacking topic.

The clinical relevance of acknowledging and predicting the amount of tissue rebound or even the time of final stability after ACL is of utmost importance, particularly in cases of esthetic rehabilitation [26]. Recent evidence demonstrates that ACL replaced flap technique leaving 3 mm between AC and GM can lead to gingival stability in a 6-month follow-up [20]. In another study with a 12-month follow-up, the mean value of the rebound was 0.3 mm, which was not statistically significant [9], demonstrating that the stability can be achieved in longer observations. However, the current available evidence regarding this topic comprises studies with short follow-up [20], small sample [9], mixed cases of CL and ACL [19], lack of correlation assessment [27], absence of papilla dimensional evaluation [28], and other factors. The absence of conclusive evidence regarding the level of post-surgery rebound and methods for achieving gingival margin stability after ACL surgery poses challenges for clinicians. The uncertainty about variables impacting the desired surgical outcome can be problematic. Post-surgical alterations in the clinical crown’s shape and size may confuse operators, and the potential modification of the periodontal phenotype introduces a novel factor that could influence future oral research and treatments. Consequently, it is crucial to investigate the procedure’s impact on esthetics and biology and emphasize the necessity for meticulous treatment planning.

The primary objective of the present study was to evaluate the stability of periodontal dimensions from baseline (T_0_) to 3 months (T_3_), 6 months (T_6_), and 12 months (T_12_) after esthetic crown lengthening in patients with excessive gingival display caused by altered passive eruption. More specifically, we aimed to assess dimensional changes in the position of the GM, clinical crown length (CCL), interdental papilla width (PW), interdental papilla height (PH), and probing depth, in central incisors, lateral incisors, and canines, and furthermore, to evaluate dimensional changes in supracrestal gingival tissue thickness (GT), distance between alveolar crest and cementoenamel junction, distance between alveolar crest and gingival margin, and alveolar crest width, by comparing tomographic measures at T_0_ and T_12_. As secondary objective, we aimed to determine the existence of a correlation between tissue rebound and the anatomical variables, such as dimensional changes in the position of the GM, CCL, PW, PH, and PD, between T_0_ and T_12_. To achieve those objectives, we aimed to comprise photographic, tomographic, and clinical assessment to evaluate diagnosis, measurements, and stability.

## Material and methods

### Study design and population

This prospective study was conducted in the Graduate Specialty Course in Periodontics Clinic, University of Brasília (UnB)—Brazil, approved in the Research Ethics Committee at the Faculty of Health Sciences of UnB (CAAE: 88,468,618.9.0000.0030), and it was conducted in accordance with the Helsinki Declaration. The sample recruitment started in January 2017, and it was finished in December 2018. Individuals were considered if they had complaints about excessive gingival display, altered passive eruption, gummy smile, or short teeth in anterior maxillary teeth, and were referred from the undergraduate dentistry clinic of UnB, the center of specialties of the University Hospital of Brasília, or private practice dentistry clinics. All patients who agreed to participate in this study after verbal and written detailed instructions, signed an Informed Consent Form to undergo the procedure of interest, and an agreement to have their photographs shared in publications, were eligible to participate on the research.

The diagnostic of APE was performed through the use of soft tissue protocol of cone-beam computed tomography (ST-CBCT), in which a lip retractor was used to allow visualization of height and thickness of gingival tissue [29]. This diagnostic method was chosen due to its high precision, low invasiveness, no need for anesthesia, and standardization of the measurement site. The condition was diagnosed if the patient presented more than two anterior maxillary teeth with the distance between GM and CEJ equal or greater than 2 mm in healthy tissue [30]. In the clinical diagnosis, an inadequate proportion between height and width of the maxillary anterior teeth was observed, in which all patients had short clinical crowns, which appeared to be square in shape⁠. The periodontal phenotype was assessed based on the gingival thickness and bone morphotype, using the ST-CBCT. Phenotype was classified as “thin” if the thickness of the gingiva was less than 1 mm and as “thick” if it exceeded 1 mm [23].

Individuals were considered in the study if they met the following inclusion criteria: (a) adults over 18 years old; (b) Visible Plaque Index (VPI) and Gingival Bleeding (GB) ≤ 10% [31]; (c) absence of fixed orthodontic device; (d) no periodontal attachment loss or history of periodontal disease; (e) no history of tooth loss in the upper anterior region; and (f) absence of diastema. Exclusion criteria comprised (a) smokers; (b) systemic contraindications for oral surgical procedures; (c) use of drugs that induce gingival hyperplasia; (d) prosthetic elements in upper anterior teeth; (e) preceding surgery in the same area of interest; (f) insufficient keratinized gingiva; and (g) pregnant or lactating individuals. The sample size was based on the number of individuals who attended the university clinics or were referred within the period of recruitment. The final sample was based on a prior study which evaluated a similar topic [20].

The sample size calculation was determined based on the primary study outcome, which focuses on evaluating changes in the GM at the 12-month mark. Employing a two-sided test with a 5% alpha risk and a 20% beta risk, it was determined that a total of 20 patients were needed to detect a statistically significant difference of ≥ 0.25 mm.

### Periodontal procedures

All included patients were invited to the Graduate Specialty Course in Periodontics Clinic (UnB) 21 days before the surgical procedure. Individuals underwent prophylaxis, dental scaling, root planing, and oral hygiene instructions. Another invitation was done 2 days before the surgical procedure in order to clinically confirm satisfactory plaque control, absence of gingival bleeding, and to reiterate all the surgical instructions. ACL surgeries were performed by periodontics graduate students under experienced periodontists’ supervision. Before surgery, patients were instructed to rinse 0.12% chlorhexidine (CHX) gluconate (PerioGard®, Colgate®, Brazil) for 60 s. Local anesthesia was administered in which the anterior and middle superior alveolar and nasopalatine nerves were blocked; also, supraperiosteal infiltration was done. The desired teeth length was marked with the Castroviejo caliper at the zenith of each tooth from the left first molar to the right first molar. An internal beveled incision was done with a 15C scalpel blade, and an intrasulcular incision was done before the removal of the demarcated gingival collar. A mucoperiosteal flap was lifted in the vestibular area and a split-flap in the interproximal papilla site. Osteoplasty was performed using high rotation spherical drills 3018 and 1016 under saline irrigation. At osteotomy, the distance from the cement-enamel junction to the bone crest was surgically established at an average of 2 mm. Subsequently, the flap was replaced with a vertical mattress suture with a nylon monofilament polyamide suture 5–0 reverse cutting needle 15 mm 3/8, considering the planned tooth length and the distance between the cement-enamel junction and bone crest. At the end of the surgery, the postoperative instructions were informed, and the prescription for medications were given to the patient. Dexamethasone (4 mg) was administered 1 h before the surgical procedure to prevent post-operative pain and swelling [32, 33]. While Dexamethasone is associated with side effects and adverse reactions in cases of prolonged medical use, a systematic review has indicated that its preemptive administration in low doses during periodontal surgeries did not exhibit any side effects [32]. Post-operatively, Amoxicillin (500 mg) was prescribed to avoid any potential local infection [19], and Ibuprofen (600 mg) was also prescribed, if the patient felt any pain [20, 34]. The administration of these medications was considered necessary due to the collaborative clinical setting in which the surgeries were conducted in a teaching hospital. Furthermore, surgeries were performed by graduate students. Consequently, a precautionary approach was adopted to mitigate the potential occurrence of post-surgical complications or infection. No patient reported drug-related adverse events. Patients returned after 14 days for suture removal, plaque control, and oral hygiene instruction.

### Data collection

Patients were requested to attend the Periodontics Clinic (UnB) at T_1_, T_3_, T_6_, and T_12_ post-operatively by means of data collection. At all follow-up periods, individuals underwent plaque control treatment and oral hygiene instructions if needed. Periodontal clinical assessment was performed by one examiner (C.A.S.A.) using a manual periodontal probe (PCP-UNC 15, Hu-Friedy, Chicago, IL, USA). The probing depth (PD) was measured at the zenith of each tooth at T_0_, T_3_, T_6_, and T_12_.

**Fig. 1:**
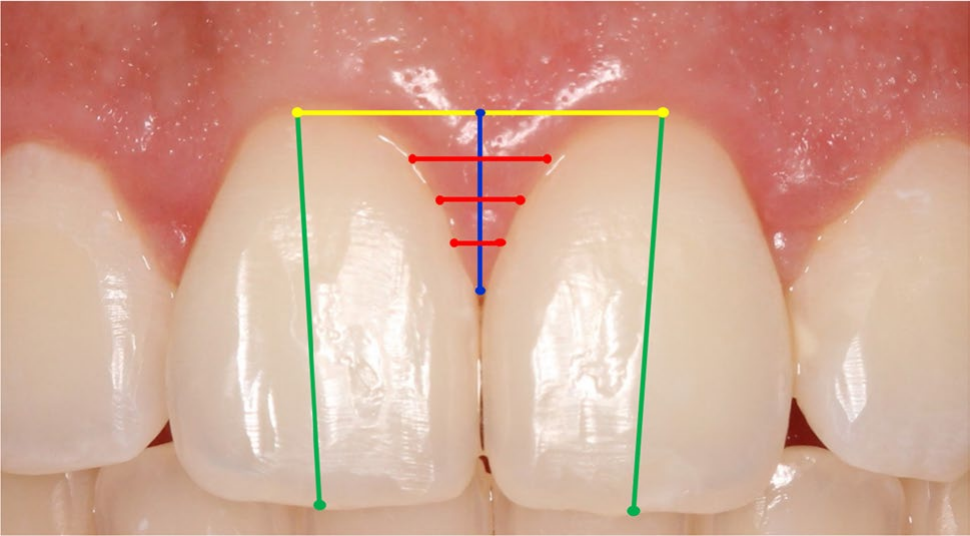
The variables collected were the clinical crown length (green), interdental papilla height (blue), and interdental papilla width subdivided into three equidistant fractions (red). For such measurements, the positions of the gingival zeniths (yellow) were used

A photographic protocol was executed at baseline and follow-up periods by the same trained and calibrated examiner (C.A.S.A.) using a Canon professional camera, 100 mm macro lens, and circular flash. Patients were lying in a horizontal position, with a lip retractor, arches occluded, and the examiner took the pictures in a frontal view and parallel to the occlusal plane [9]. All the image files were transferred to the ImageJ software (National Institutes of Health, EUA, ImageJ version v1.51j8) where they were analyzed by one trained and calibrated examiner (M.U.M.). Measurements were done twice by the same examiner, with an interval of 15 days, and the average values of the two measurements were used for analysis. To calibrate the software regarding each image, the examiner drew a line from the most distal point of the right central incisor to the most distal point of the left central incisor. This line was then converted into millimeters considering the exact same clinical measurement. Image files of T_0_, T_3_, T_6_, and T_12_ were used to measure the following parameters (Fig. 1):

1. Clinical crown length (CCL): a line was drawn starting on the tooth zenith and extending to the most central point of the incisal edge in CI, LI, and CN group of teeth.
  - Considering that photograph assessment was not possible due to bleeding and suture, this was the only variable measured immediately after surgery (T_1_). A Castroviejo caliper was used to measure CCL using the same parameters of the photograph protocols.
2. Interdental Papilla height (PH): at first, a line was drawn from one zenith tooth to his adjacent zenith tooth. Then, a vertical line was drawn from the papilla point site to the horizontal line in three groups central papilla (CP), central-lateral papilla (CLP), and lateral-canine papilla (LCP).
3. Interdental Papilla width (PW): the PH line was divided into three equidistant horizontal lines being a superior, median, and an inferior line.

Cone-beam computed tomography scans were performed with an i-CAT tomograph (Imaging Sciences International, Inc., Hatfield, PA, USA), and the images were acquired with the Vision software. Patients were seated with their chin and head stabilized. A capture of the maxilla was taken (dimensions 6 × 17 cm) for 40 s with the following programming: voxel size of 0.2 mm; grayscale: 14 bits; focal point: 0.5 mm; image detector: amorphous silicon flat panel; image acquisition: one rotation of 360°. Images were generated in XORAN files and saved in DICOM format.

**Fig. 2:**
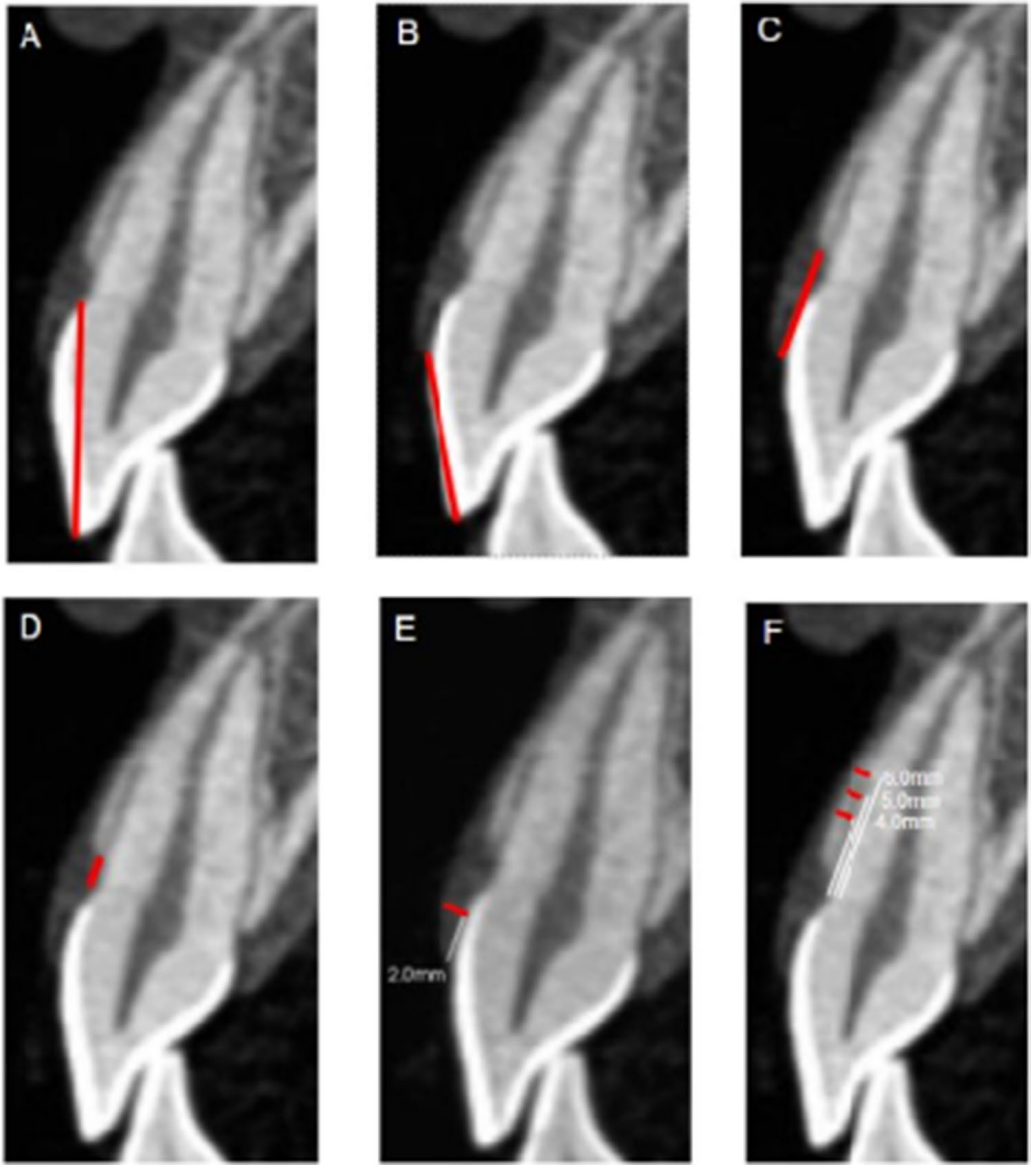
Tomographic image of tooth 11 showing structures measurement (red). Anatomical crown length (**A**); clinical crown length (**B**); distance between the alveolar crest and the gingival margin (**C**); distance between the alveolar crest and the cementoenamel junction (**D**); gingival thickness (**E**); bone thickness (**F**)

Thus, the pre- (T_0_) and post-surgical (T_12_) tomographic data of each patient were recorded using the CS 3D Imaging Software program and imported into an Excel spreadsheet. Teeth were analyzed from cross-sections images placed after the long axis of the tooth, in such a way that the entire endodontic canal could be seen. Contrast effect was used to refine and enhance the image to ensure the accuracy of each measurement. All measurements were taken by a single researcher trained to interpret tomographic images (A.M.S.G.) and were performed twice within a 15-day interval, so that an intra-examiner calibration assessment was possible. The following measurements were made on each tomographic image (Fig. 2):

A. Anatomical Crown length (tAC).
B. Clinical Crown length (CCL)
C. Distance between the alveolar crest and the gingival margin (AC-GM).
D. Distance between the alveolar crest and the cementoenamel junction (AC-CEJ).
E. Gingival Thickness (GT): measured at 2 mm from the GM level.
F. Bone Thickness (BT): measured at three different levels; at 4, 5, and 6 mm apical from the CEJ.

### Statistical analysis

Data were submitted to statistical analysis by using the SPSS statistical software, version 24.0 (IBM Corp. Released 2016. IBM SPSS Statistics for Windows, Version 24.0. Armonk, NY: IBM Corp.). Preliminary, Shapiro–Wilk normality test failed in the data distribution verification. Comparisons of CCL, PD, GM, PW, and PH, among different follow-up times, were performed by Kruskal–Wallis test, followed by a post hoc Dunn test with Bonferroni correction for pair-wise comparisons when differences were statistically significant. Tomographic measures, such as tAC, AC-GM, AC-CEJ, BT, and GT, were compared by Wilcoxon signed rank test according to the time of follow-up. To determine the correlation between the variables included in the study, Spearman’s correlation test was applied [35]. All tests were performed at 5% of level of significance (*p* < 0.05).

To calculate the method error for the measurements, all variables were submitted to a second measurement 15 days after the first one. To detect random error, the formula recommended by Dahlberg was used: *E*2 = Σ*d*2/2*n*, where “*E*” indicates the error to be calculated, “*d*” indicates the difference between the two measurements for the same quantity, and “*n*” indicates the number of pairs of images compared [36]. To detect systematic error, Student’s *t* test for paired data was used, comparing the values obtained in the first and second measurements. To calculate the measurement error, we did not discriminate between the deviated side in relation to the contralateral side; only the measurements between the right side and the left side were considered.

## Results

Twenty individuals participated in this study for the 12-month follow-up period, considering all the sample completed the follow-up without dropouts. The sample was composed of 100% female patients, with a mean age of 25.95 ± 2.77 (20–32) years. The patients were systemically healthy, non-smokers, not using continuous medication, and had no contraindications for oral surgery or postoperative medication. The surgical procedures were successful, and the patients had no postoperative complications. A total of 120 teeth were included in the analyses, being three equal numbers of CI, LI, and CN. Regarding the interdental papillae, 100 sites were assessed, and they were divided into three groups of CP (20), CLP (40), and LCP (40). The Dahlberg error ranged from 0.01 to 0.19 mm across all variables, with no statistically significant differences observed between the initial and subsequent measurements for any of them.

Photographic and clinical measurements descriptive statistics regarding CCL in T_0_, T_1,_ T_3_, T_6_, and T_12_ are presented in Table 1. Before ACL surgery, CI presented a CCL of 7.95 ± 0.92 mm, reaching 10.34 ± 0.47 mm at the time of suture (*p* < 0.05). From T_1_ to T_3_, a non-statistically significant rebound was found as the number downsized to 9.99 ± 0.73 mm (*p* > 0.05). From T_3_ to T_12_, the non-statistical difference in the rebound progression was found, considering that the mean CCL in T_12_ was 9.59 ± 0.87 mm (*p* > 0.05). Lateral incisors showed a similar statistical difference between T_0_ and T_1_ being 6.73 ± 0.82 mm and 8.49 ± 0.71 mm, respectively (*p* < 0.05). However, those numbers remained relatively stable in T_3_, T_6_, and T_12_ follow-up periods, being 8.51 ± 0.73 mm, 8.37 ± 0.69 mm, and 8.22 ± 0.73 mm, respectively (*p* > 0.05). Canines showed similar outcomes regarding stability compared to CI. In baseline, the mean CCL was 7.58 ± 0.68 mm, and it statistically increased to 9.62 ± 0.57 mm at suture time (*p* < 0.05). No statistical difference was found comparing T_1_ and T_3_, despite the number downsizing to 9.16 ± 0.55 mm (*p* > 0.05). From T_3_ to T_12_, the CCL showed stability, considering that the length at T_12_ was 8.99 ± 0.51 mm (*p* > 0.05).

**Table 1:** Photographic, tomographic, and clinical data related to dental and periodontal measurements (in millimeters) from baseline and follow-up periods

	*Follow-up*	*CI*	*LI*	*CN*	*Mean value*
*tAC*	T_0_	10.79 ± 0.83^(A)^	9.47 ± 0.83^(A)^	9.79 ± 0.54^(A)^	10.02 ± 0.65^(A)^
	T_12_	10.81 ± 0.75^(A)^	9.56 ± 0.73^(A)^	9.99 ± 0.56^(A)^	10.12 ± 0.57^(A)^
*AC-GM*	T_0_	4.14 ± 0.65^(A)^	4.06 ± 0.67^(A)^	3.42 ± 0.49^(A)^	3.87 ± 0.49^(A)^
	T_12_	3.49 ± 0.46^(B)^	3.73 ± 0.56^(A)^	3.28 ± 0.54^(A)^	3.50 ± 0.44^(B)^
*AC-CEJ*	T_0_	1.36 ± 0.30^(A)^	1.48 ± 0.46^(A)^	1.34 ± 0.54^(A)^	1.39 ± 0.34^(A)^
	T_12_	2.11 ± 0.36^(B)^	2.19 ± 0.45^(B)^	2.13 ± 0.56^(B)^	2.15 ± 0.35^(B)^
*GT*	T_0_	1.27 ± 0.17^(A)^	1.17 ± 0.18^(A)^	1.16 ± 0.17^(A)^	1.20 ± 0.13^(A)^
	T_12_	1.73 ± 0.21^(B)^	1.38 ± 0.16^(B)^	1.35 ± 0.22^(B)^	1.49 ± 0.16^(B)^
*BT*	T_0_	1.00 ± 0.33^(A)^	0.95 ± 0.33^(A)^	1.11 ± 0.43^(A)^	1.02 ± 0.33^(A)^
	T_12_	0.86 ± 0.20^(A)^	0.77 ± 0.20^(B)^	0.82 ± 0.19^(B)^	0.82 ± 0.15^(B)^
*CCL*	T_0_	7.95 ± 0.92^(A)^	6.73 ± 0.82^(A)^	7.58 ± 0.68^(A)^	7.42 ± 0.70^(A)^
	T_1_	10.34 ± 0.47^(B)^	8.49 ± 0.71^(B)^	9.62 ± 0.57^(B)^	9.48 ± 0.49^(B)^
	T_3_	9.99 ± 0.73^(B)^	8.51 ± 0.73^(B)^	9.16 ± 0.55^(B)^	9.23 ± 0.62^(B)^
	T_6_	9.75 ± 0.84^(B)^	8.37 ± 0.69^(B)^	9.04 ± 0.45^(B)^	9.03 ± 0.61^(B)^
	T_12_	9.59 ± 0.87^(B)^	8.22 ± 0.73^(B)^	8.99 ± 0.51^(B)^	8.93 ± 0.65^(B)^
*PD*	T_0_	1.98 ± 0.57^(A)^	1.86 ± 0.60^(A)(D)^	1.61 ± 0.50^(A)^	1.81 ± 0.52^(A)^
	T_3_	1.27 ± 0.32^(B)^	1.18 ± 0.42^(B)(C)^	1.21 ± 0.36^(B)^	1.23 ± 0.33^(B)^
	T_6_	1.25 ± 0.24^(B)^	1.14 ± 0.27^(B)^	1.23 ± 0.28^(B)^	1.21 ± 0.22^(B)^
	T_12_	1.62 ± 0.50^(A)(B)^	1.49 ± 0.42^(C)(D)^	1.68 ± 0.39^(A)^	1.60 ± 0.38^(A)^
	***Follow-up***	***CP***	***CLP***	***LCP***	***Mean value***
PH	T_0_	3.55 ± 0.66^(A)^	2.97 ± 0.50^(A)^	3.01 ± 0.40^(A)^	3.17 ± 0.38^(A)^
	T_3_	5.05 ± 0.73^(B)^	4.26 ± 0.51^(B)^	3.84 ± 0.37^(B)^	4.38 ± 0.47^(B)^
	T_6_	4.89 ± 0.63^(B)^	4.21 ± 0.51^(B)^	3.94 ± 0.38^(B)^	4.35 ± 0.45^(B)^
	T_12_	4.72 ± 0.64^(B)^	4.12 ± 0.47^(B)^	3.88 ± 0.40^(B)^	4.24 ± 0.42^(B)^
PW	T_0_	2.88 ± 0.85^(A)^	2.39 ± 0.36^(A)^	1.89 ± 0.26^(A)^	2.39 ± 0.45^(A)^
	T_3_	2.78 ± 0.34^(A)^	2.49 ± 0.30^(A)^	2.15 ± 0.26^(B)^	2.47 ± 0.19^(A)(B)^
	T_6_	2.82 ± 0.49^(A)^	2.51 ± 0.28^(A)^	2.13 ± 0.17^(B)^	2.49 ± 0.21^(A)(B)^
	T_12_	2.98 ± 0.62^(A)^	2.60 ± 0.34^(A)^	2.18 ± 0.21^(B)^	2.58 ± 0.31^(B)^

Central incisors (CI); Lateral incisors (LI); Canines (CN); Anatomical Crown length (tAC); Alveolar crest and the gingival margin (AC-GM); Alveolar crest and the cementoenamel junction (AC-CEJ); Gingival thickness (GT); Bone thickness (BT); Clinical crown length (CCL); Probing depth (PD); Interdental papilla height (PH); Interdental papilla width (PW); Baseline (T_0_); Immediate post-operative (T_1_); 3 months (T_3_); 6 months (T_6_); 12 months (T_12_); Central papilla (CP); Central-lateral papilla (CLP); Lateral-canine papilla (LCP)

(A), (B), (C), and (D) indicate statistical difference between follow-up variables (*p* < 0.05)

**Fig. 3:**
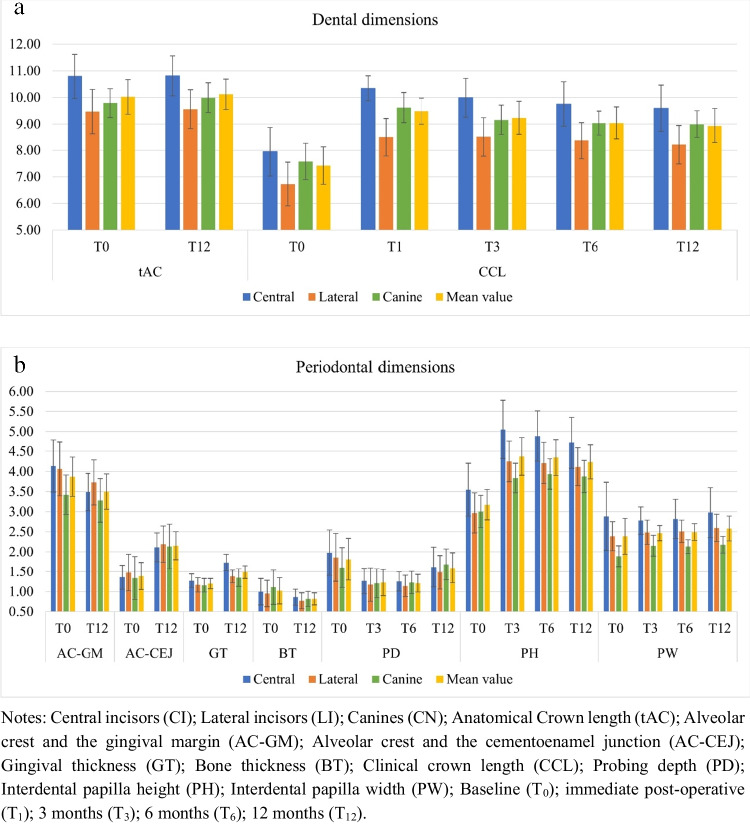
Photographic, tomographic, and clinical data related to (**a**) dental dimensions and (**b**) periodontal dimensions (in millimeters) from baseline and follow-up periods

Descriptive data regarding the height and width of interdental papilla at T_0_, T_3_, T_6_, and T_12_ follow-up periods are shown in Table 1 and Fig. 3. All groups of interdental papillae showed similar progression in PH, considering the statistical difference between T_0_ and T_3_ (*p* < 0.05), and dimensional stability between T_3_ and T_12_ (*p* > 0.05). A different pattern was found in the PW statistical analysis regarding the groups. The PW of CP area was 2.88 ± 0.85 mm in baseline and 2.98 ± 0.62 mm at T_12_ (*p* > 0.05). CLP sites presented similar results as the papilla remained dimensionally stable between T_0_ and T_12_ (*p* > 0.05). A different pattern was found in LCP regions, as the papilla increased its width from T_0_ to T_3,_ where the numbers were respectively 1.89 ± 0.26 mm and 2.15 ± 0.26 (*p* < 0.05). Values of T_6_ and T_12_ remained stable when compared to the previous period, as the means were 2.13 ± 0.17 mm and 2.18 ± 0.21 mm, respectively (*p* > 0.05). The measures accounting for all group of interdental papillae showed that there was a statistically significant increase in PW from T_0_ to T_12_, which were respectively 2.39 ± 0.45 mm and 2.58 ± 0.31 mm.

Table 1 and Fig. 3 also show the descriptive values of tAC size at T_0_ and T_12_, showing that there was no statistically significant difference between the measurements of the two periods, as expected.

Before the ACL, the mean values for the AC-GM distance in all teeth were 3.87 ± 0.49 mm and, at T_12_, it corresponded to 3.50 ± 0.44 mm and, for CI, it was from 4.14 ± 0.65 to 3.49 ± 0.46 mm, which revealed a significant difference (*p* < 0.05). However, for canines and lateral incisors, there was no significant difference. In the AC-CEJ distance, a significant difference (*p* < 0.05) was found in all teeth between T_0_ (1.39 ± 0.34 mm) and T_12_ (2.15 ± 0.35 mm). The three groups of teeth presented similar results and reached a statistically significant difference (*p* < 0.05), as described in Table 1.

Table 1 and Fig. 3 also present the descriptive statistics of the measurements related to GT and BT obtained by ST-CBCT image at T_0_ and T_12_. Statistical analysis of the GT revealed a thickening of the gingiva after the ACL. Thickness increased on average for all teeth and individually for the three groups of teeth. Before the surgical procedure, all teeth had an average of 1.20 ± 0.13 mm; at T_12_, they reached 1.49 ± 0.16 mm. The CN and the LI had similar values and statistics between T_0_ and T_12_, being 1.16 ± 0.17 to 1.35 ± 0.22 mm in the CN, and for the LI, from 1.17 ± 0.18 to 1.38 ± 0.16 mm. CI showed greater gingival thickening between T_0_ and T_12_, with 1.27 ± 0.17 to 1.73 ± 0.21 mm. For the BT, there was a statistical difference between the two periods analyzed for the average of all teeth, being 1.02 ± 0.33 mm before the ACL and 0.82 ± 0.15 mm at T_12_. CN and LI had similar statistical results, showing a reduction in bone thickness, which is in line with the osteoplasty performed during the procedure. Values were 1.11 ± 0.43 mm and 0.95 ± 0.33 mm at T_0_, and at T_12_, 0.82 ± 0.19 mm and 0.77 ± 0.20 mm, respectively. However, the CI did not present a statistically significant difference, as shown in Table 1.

Clinical crown length at baseline presented a strong positive correlation with PH at T_0_ (*r* = 0.71*), and moderate correlation with CCL at T_1_ (*r* = 0.68*), CCL at T_12_ (*r* = 0.62*), and tAC (*r* = 0.49*). CCL at T_1_ showed a moderate and positive correlation with CCL at T_12_ (*r* = 0.62*), tAC (*r* = 0.48*), AC-CEJ at T_12_ (*r* = 0.55*), and BT at T_12_ (*r* = 0.56*). CCL at T_12_ also presented a moderate positive correlation with PH at T_0_ (*r* = 0.54*), PW at T_0_ (*r* = 0.47*), PW at T_12_ (*r* = 0.68*), tAC (*r* = 0.67*), and BT at T_12_ (*r* = 0.55*). Inversely, the correlation analysis showed that the larger the PD at T_0_ the smaller the CCL at T_12_ (*r* =  − 0.56*). The larger the PH at T_0_, the smaller the PD also at T_0_ (*r* =  − 0.54*). The larger the PW at T_0_ the larger it will be at T_12_ (*r* = 0.58*). PW at T_12_ presented a strong positive correlation with tAC (*r* = 0.71*) and moderate correlation with AC-GM at T_12_ (*r* = 0.47*), and a negative correlation with AC-CEJ at T_0_ (*r* =  − 0.60*). A few other negative correlations were found between PD at T_0_ and BT at T_12_ (*r* =  − 0.63*), tAC and AC-CEJ T0 (*r* =  − 0.52*). GT at T_0_ presented positive correlation with GT at T_12_ (*r* = 0.65*), AC-CEJ at T_12_ (*r* = 0.48*), AC-GM at T_12_ (*r* = 0.49*), and BT at T_0_ (*r* = 0.60*). Some other important positive correlations were found between BT at T_0_ and GT at T_12_ (*r* = 0.62*), and BT at T_0_ and AC-GM at T_12_ (*r* = 0.52*).

## Discussion

The present prospective clinical study was performed to evaluate the dimensional stability of periodontal tissues such as gingival margin, probing depth, and interdental papilla 12 months after esthetic crown lengthening surgical procedure in patients with altered passive eruption. The results showed a non-statistically significant rebound of 0.55 mm in all groups of teeth when the desired crown length (T_1_) is compared to the achieved crown length at 12 months (T_12_). The rebound was higher in CI with a mean of 0.75 mm and CN with 0.63 mm. Lateral incisors showed more stability through the follow-up period since they did not demonstrate a high difference between T_1_ and T_12_, with an average of 0.27 mm which is not clinically significant. Furthermore, interdental papilla height statistically increased due to ACL, and this value remained stable for 3, 6, and 12 months. Papilla width presented increased values when the baseline period was compared to the end of the follow-up period, demonstrating a statistically significant enlargement of the papilla during the healing process for all groups of teeth together. An interesting negative correlation was found between the PD at baseline and CCL at T_12_, confirming that the shorter the PD before the surgery, the greater the CCL will be at the end of the follow-up period.

### Gingival margin (GM) and clinical crown length (CCL)

The main purpose of performing an esthetic crown lengthening is granting a new size to the clinical crown at the end of the surgery and achieving the planned amount at the end of the healing period [20]. However, a rebound happens in cases in which the gingival margin migrates from its sutured position to a coronal direction of the teeth, making the crown to be clinically shorter than planned [17]. One of the main causes of the lack of stability of CCL seems to be the unsatisfactory AC-GM distance left at the suturing moment [15]. Post-surgical distance of 3 mm has shown more stable progress, considering that distances such as ≤ 2 mm usually lead to greater rebound and ≥ 4 mm are associated with gingival recession [17, 20, 37]. Taking this gold standard precedent into consideration, the AC-GM distance was 3 mm in all individuals of the present study.

Outcomes of a pre-restorative clinical crown lengthening study have suggested that the rebound starts its process 1 month after surgery, showing meaningful progression until 12 months of follow-up. In buccal/lingual sites the AC-GM measures at baseline, end of the surgery, 1 month, and 12 months were respectively 1.6 ± 2.5 mm, 5.7 ± 2.4 mm, 4.1 ± 2.3 mm, and 2.8 ± 2.6 mm [14]. Another study showed different results regarding the duration of rebound through the healing process, since a stability was found from 42 days to 6 months of follow-up in individuals where the distance was left at 3 mm [20]. The study was focused on patients with APE and the surgery was done for esthetic reasons, and the amount of rebound was 0.94 ± 0.53 mm, 0.10 ± 0.28 mm, and − 0.26 ± 0.40 mm in individuals whose AC-GM distance was respectively < 2 mm, 3 mm, and > 4 mm. However, the study did not follow the healing progress from 6 to 12 months postoperatively. More stable outcomes were found in another ACL study, in which authors found no statistical difference comparing results after suture with 6 months of observation, even though a rebound of 0.4 mm was reported [38]. In a 12-month follow-up study, two different interventions were considered, being a (1) two-stage crown lengthening and a (2) two-stage crown lengthening [13]. In both groups, 80% of patients were within ± 0.5 mm respective to the planned dimension of CCL. Individuals of the present study showed a rebound of 0.75 mm in CI and 0.71 mm in CN, what shows a diversity in outcomes within publications using similar surgical techniques.

### Probing depth (PD)

Gingival sulcus depth is occasionally used as a diagnostic criterion in individuals with altered passive eruption. Some authors claim that a probing depth greater than 3 mm is an indicative of APE in areas without periodontal disease or inflammation [10], even though this concept was proven to be obsolete since APE patients showed a mean PD of 1.5 mm and 1.97 mm depending on the study [20, 24]. In the present study, a probing depth of 1.81 ± 0.52 mm was found in baseline, and this value showed a negative moderate correlation (*p* < 0.05) with the clinical crown length at the 12 months. This outcome enriches the concept of short clinical crowns having inverse correlation to a deep gingival sulcus.

Several healing processes related to soft tissues take place after ACL surgical procedure. The gingival margin rebound evaluation should take into consideration how each supracrestal gingival tissue evolves post-operatively, so it is possible to address which structure is responsible for the rebound. The difference in the dentogingival unit between patients with APE and patients without APE is directly related to the fact that those individuals have longer supracrestal gingival tissue, comprised by a long junctional epithelium, short connective tissue attachment, and a normal gingival sulcus [8, 24]. Studies suggested that the coronal migration of GM happens during the establishment of the new gingival sulcus, meaning that the supracrestal gingival structures accountable for the rebound are the sulcular/junctional epithelium and marginal gingiva [14, 27]. A study evaluating postoperative changes in the PD after ACL in patients with APE reported that canines, lateral incisors, and central incisors had a statistically significant decrease between baseline and 42 days after surgery [20]. From 42 days until 6 months post-operatively, this PD showed stability (*p* > 0.05). However, when the analysis included first and second premolars, there was no statistical difference regarding any follow-up period compared to baseline. The study reported a PD of 1.97 mm at baseline, 1.57 mm at 42 days, and 1.86 mm at 6 months of follow-up for all groups of teeth. The authors concluded that CN, LI, and CI had a decrease in the PD, and this value remained stable postoperatively. A similar study with the same follow-up showed equivalent outcomes, since the same group showed a statistically significant decrease in PD from 2.1 mm in baseline to 1.1 mm at 6 months of follow-up [38]. In the present study, the PD at baseline was 1.81 ± 0.52 mm, significantly decreased to 1.23 ± 0.33 mm at 3 months and increased to 1.60 ± 0.38 mm at the end of the follow-up period, showing no statistical difference in regards the PD in the baseline. Those outcomes confirm that, after ACL, the PD tends to return to its original length [14]. It is important to state that the gingival sulcus is non-existent (or 0.00 mm) immediately after surgery. One concept indicates that the coronal migration of the GM happens in order to create a sulcus, since there is simultaneously a PD growth and a CCL shortening [27].

### Interdental papilla height (PH) and width (PW)

The interdental papilla preoperative assessment is one important diagnostic criterion regarding APE condition. In individuals with APE, a disproportionate rate between width and height can be found on the papilla, together with other clinical observations, such as short clinical crown and deep probing depth [7, 24]. Different techniques have been evidenced in the past decades regarding papilla interdental management in ACL procedures [39]. Evidence suggests that the papilla should be disturbed as little as possible to avoid rebound or black spaces [40]. Also, intending not to block blood supply during the healing period, it is important to perform a split-thickness flap and to be careful during incision in the area. Removal of interproximal bone should not reach the papilla sites, since they are sensitive regarding dimensional changes [39–41].

During ACL 12 months post-operatively, supracrestal soft tissue grows coronally in order to create new gingival sulcus and interdental papilla [27]. When this growth happens in the gingival margin, a classic rebound is found. However, when soft tissue growth is found in the mesiodistal direction, a dimensional change in the PW is present. In this case, the consequences are more related to the shape of the teeth instead of the clinical crown length. If there is an increase in the PW, the clinical crown becomes more triangular than planned. Our study showed a statistically significant increase in the PH in all follow-up periods compared to baseline. This increase demonstrates that the disproportion between PW and PH was corrected as a consequence of the surgical procedure. However, the PW showed an unexpected behavior, since there was an increase in all groups of teeth comparing T_12_ to T_3_. The clinical repercussion of this dimensional change was a difference in the final clinical crown shape, which was presented as more triangular. In this case, if the patient agrees, a two-stage procedure could be performed to achieve a better gingival scaffold [13]. However, the difference between PW at T_3_ and T_12_ was only 0.2 mm, which might not be clinically significant in most cases.

### Tomographic analysis

The present study also compared, by means of tomographic images, the changes in periodontal tissues of individuals in two stages: T_0_ and T_12_. Statistical analyses showed that, for the size of the tAC, there was no significant difference in all teeth (*p* > 0.05), which indicates the reliability of the data recorded by the tomographic images and the measurements. The absence of statistical difference serves to ratify the calibration of the researcher responsible for the two tomographic measurements of each variable, carried out with a space of 15 days between them. Furthermore, the accuracy of measurements also points to the differences across time not being due to possible errors in calibration.

With regard to the AC-GM distance, the LI and CN did not present a statistically significant difference (*p* > 0.05) between T_0_ and T_12_. However, for CI and all teeth group, the outcome was different—a statistically significant decrease was found between T_0_ and T_12_ (*p* < 0.05). These results indicate that the supracrestal gingival tissues tend to return to their initial dimensions after the ACL. The surgical protocol established that the flap should be sutured approximately 3 mm apart from the AC, so that the supracrestal soft tissue length should be reestablished and reduces the chances of a rebound during the repair period, which is in agreement with previous studies that obtained satisfactory results [42, 43]⁠. Thus, we hypothesized that there was no statistically significant difference for the CN and LI because their mean AC-GM distance was closer to 3 mm at T_0_ (Table 2), which is the value considered standard in a patient without APE [22, 44]. A study with a sample of 30 patients pointed out that, during a 12-month follow-up period after crown lengthening for restorative purposes, the GM showed a tendency to grow in a coronal direction from the level defined at surgery [45]⁠. On the other hand, more recent studies reported that a coronal displacement from the GM position could be expected within period of approximately 3 months after surgical crown lengthening, and that the original dimension of the supracrestal insertion will be restored after 6 months [42, 43]⁠. These observations illustrate that divergences in esthetic treatment findings can arise as a result of variability in tissue response to surgical trauma or the technique used [15].

**Table 2:** Spearman correlation between dental and periodontal measurements at baseline and 12-month follow-up

	CCL T_0_																	
CCL T_1_	0.68*	CCLT_1_																
CCL T_1_–T_12_	− 0.27	0.20	CCL T_1_–T_12_															
CCL T_12_	0.62*	0.62*	− 0.53*	CCL T_12_														
PH T_0_	0.71*	0.39	− 0.36	0.54*	PH T_0_													
PH T_12_	0.08	0.10	− 0.48*	0.40	0.26	PH T_12_												
PWT_0_	0.26	0.33	− 0.09	0.47*	0.12	− 0.01	PW T_0_											
PW T_12_	0.14	0.32	− 0.40	0.68*	0.09	0.21	0.58*	PW T_12_										
PD T_0_	− 0.44	− 0.29	0.32	− 0.56*	− 0.54*	− 0.24	− 0.30	− 0.21	PD T_0_									
PD T_12_	0.07	0.22	− 0.05	0.23	− 0.19	− 0.32	− 0.07	0.00	− 0.05	PD T_12_								
tAC	0.49*	0.48*	− 0.36	0.67*	0.23	0.22	0.37	0.71*	− 0.22	0.03	tAC							
AC−GM T_0_	− 0.16	0.38	0.26	0.07	− 0.35	0.07	0.07	0.31	0.10	0.07	0.16	AC−GM T_0_						
AC-GM T_12_	0.17	0.36	− 0.01	0.28	− 0.01	0.12	0.04	0.47*	0.05	− 0.23	0.69*	0.50*	AC-GM T_12_					
GT T_0_	0.33	0.31	0.06	0.06	− 0.18	0.03	− 0.01	0.00	0.14	0.06	0.33	0.23	0.49*	GT T_0_				
GT T_12_	0.29	0.35	− 0.25	0.35	− 0.03	0.17	0.04	0.30	0.18	0.32	0.51*	0.36	0.39	0.65*	GT T_12_			
AC-CEJ T_0_	− 0.05	0.08	0.45	− 0.45	− 0.01	− 0.31	− 0.30	− 0.60*	0.28	0.00	− 0.52*	0.28	− 0.11	− 0.02	− 0.08	AC-CEJ T_0_		
AC-CEJ T_12_	0.27	0.55*	0.17	0.13	0.10	0.18	− 0.08	0.08	− 0.04	− 0.08	0.33	0.49*	0.60*	0.48*	0.40	0.38	AC-CEJ T_12_	
BT T_0_	0.24	0.18	− 0.42	0.32	− 0.11	0.30	0.22	0.43	− 0.09	0.08	0.61*	0.28	0.52*	0.60*	0.62*	− 0.27	0.41	BT T_0_
BT T_12_	0.34	0.56*	− 0.04	0.55*	0.20	0.20	0.34	0.32	− 0.63*	0.32	0.40	0.28	0.25	0.33	0.38	− 0.15	0.44	0.45

Clinical Crown length (CCL); Alveolar crest and the gingival margin (AC-GM); Gingival thickness (GT); Bone thickness (BT); Baseline (T_0_); Immediate post-operative (T_1_); 12 months (T_12_)

*(*p* < 0.05); ** (*p* < 0.01)

An interesting result was recently reported in which the LI and CI showed greater soft tissue rebound at T_3_ and T_6_ when compared to other types of teeth [46]⁠.⁠ It has been hypothesized that this may have occurred due to a smaller AC-GM distance left at the time of suturing. From 42 days to 6 months of follow-up, the position of the GM remained stable when the distance was 3 mm and ≥ 3.5 mm, while when AC-GM was ≤ 2 mm, a significant recurrence was observed between 3 and 6 months [46]. In addition, our study showed a positive, moderate, and significant correlation between the AC-GM dimension at T_0_ and T_12_ (*r* = 0.50, *p* < 0.05). That is, the greater the AC-GM dimension in T_0_, the greater will be that same dimension in T_12_. This confirms the premise that the supracrestal soft tissues seek to reestablish themselves after ACL, and also, that the planned CCL is more stable if the AC-GM distance at T_1_ is similar to the same structure before surgery [19, 47]⁠. Thus, it is extremely important that there is an individualized pre-surgical planning for each tooth.

Statistical analyses also showed that there was a significant difference in all teeth in the AC-CEJ distance (*p* < 0.05), indicating that the osteotomy performed during the ACL procedure remained stable after 12 months, with no tendency to return to the initial position of the alveolar bone crest. The mean amount for AC-CEJ at baseline was 1.39 ± 0.34 mm, and a mean AC-GM distance was 3.87 ± 0.49 mm also at T_0_. Thus, it was necessary to perform a total flap associated with osteotomy/osteoplasty in these cases to restore the supracrestal insertion [15, 48]. Although the biological dimensions vary and are unique to each clinical condition, other studies highlighted the importance of maintaining a distance of 2.5 mm between AC-CEJ in order to provide a supracrestal insertion of approximately 3 mm [44, 49, 50]⁠. This distance is considered healthy and physiological, and must be filled by a band of connective tissue, junctional epithelium, and gingival sulcus [50]⁠. For the patients in the present study, osteotomy was performed, in which an average AC-CEJ distance of 2.5 mm was left, and this measurement decreased to 2.15 ± 0.35 mm at T_12_, demonstrating an absence of stability of alveolar bone crest after surgery was performed. A previous study reported that when performing a follow-up radiographic evaluation for 12 months after crown lengthening surgery in premolars, it was concluded that there was no significant radiographic change in the height of the AC at the end of the period [51]⁠. However, the evaluation was done radiographically, meaning that only the interdental bone could be included in the conclusions. Therefore, the level of the alveolar crest after ACL healing can be stable, but also could show a level of rebound [22, 44, 52].

There is a scarcity of information regarding the rebound of periodontal tissues after ACL in cases for the treatment of EGD, especially when the procedure involves osteoplasty. However, the statistical analyses of the present study demonstrated that, once the osteoplasty was performed, there was a statistically significant difference in BT between T_0_ and T_12_ in all teeth (*p* < 0.05), revealing a tendency to postoperative stability. Another result in the present study was the tendency towards gingival tissue thickening, observed at time T_12_ in all three groups of teeth. This finding was surprising, since in the surgical procedure, the thickness of the tissue does not change, because the flap performed is the total flap.

There are discussions in literature about different responses according to each periodontal phenotype after ACL [53]⁠,⁠ but all patients in our sample had a thick phenotype. We used a classification published in 2022, which determines the thickness of the gingiva as “thin” when the thickness is less than 1 mm and as “thick” when greater than 1 mm, using ST-CBCT [53].⁠

There are no reports in the literature so far regarding the increase in GT after ACL, but one study reported greater instability of the GM observed in patients who had a quantity of keratinized mucosa and a thick bone pattern at baseline [25]. Factors such as patient age, periodontal phenotype, tooth type, position of the post-surgical flap, amount of bone reduction, and surgical technique can influence the maturation process [25]⁠. Zucchelli reports the findings of post-surgical change in GM to the clinical phenomenon “Creeping Attachment”, which is characterized by the migration of postoperative marginal gingival tissue in a coronal direction, in order to reform a new “physiological” supracrestal gingival unit [25]. However, this term is used for procedures where gingival grafts are performed and not ACL. However, the reason why the tissue showed a tendency to thicken is still unknown, since only the bone thickness was modified during surgery. A conjecture raised to explain this is that the tissue thickening could be related to a protective response, since the bone was thinned. However, the search for factors that may be related to the increase in tissue thickness will be highlighted in future studies. Answers to these questions may help the clinician to better delineate all stages of the surgical procedure with greater predictability, in the esthetic management of gummy smile cases.

Finally, a positive, moderate, and significant correlation was found between bone thickness and gingival thickness at baseline in the present sample (*r* = 0.60, *p* < 0.01)—meaning that the greater the buccal bone thickness of an individual, the greater the corresponding gingival thickness. This corroborates the classification of periodontal phenotypes established in the literature, in which the thick phenotype is described based on the evaluation of gingival thickness, together with bone thickness and the keratinized mucosa strip, in which the GT tends to be greater when the underlying BT is also great [15, 50]⁠⁠. This relationship between BT and GT in the upper anterior teeth has been similarly analyzed by other studies [54–56]. A study with a sample of 15 patients, which used ST-CBCT and transgingival probing, did not find a direct correlation between the tissues [54]⁠. On the other hand, another study described a moderate positive correlation between BT and GT, using parallel profile radiographs of central incisors in 60 patients [55]. On another study, after measuring the BT and GT at 2, 4, and 6 mm apical to the CEJ in ST-CBCT, concluded that the different gingival biotypes and their relationship with the BT varied around the anterior upper teeth [56]⁠. Thus, the correlation found in our analysis agrees with a part of the literature, but further studies are needed to discuss the topic.

It is also important to note that a few variables at baseline showed a moderate correlation to changes after 12 months of follow-up. The correlation between tAC and CCL (at baseline, T_1_, and T_12)_ shows that there is a proportion between anatomical and clinical crown measures. It means that the longer the tAC and CCL at baseline, the longer CCL will be at T_1_ and T_12_. The negative correlation between PH and PD at baseline also shows that the shorter the PH, the shorter the CCL, and the deeper the PD. Interestingly, the anatomical crown correlates with PW at T_12_, meaning that the longer the anatomical crown the wider the papilla will be by the end of the follow-up period. In our study, we had different values for AC-GM since each patient has a different proportion to be followed during the surgery regarding the final CCL. The positive correlation between tAC and AC-GM at T_12_ demonstrates that the longer the anatomical crown, the longer the supracrestal soft tissue. One of the results we found is related to increased GT after ACL surgery, and the correlation shows that there is a moderate positive correlation between GT at T_12_ and BT and GT at T_0_. This could indicate a tendency for supracrestal soft tissue overgrowth when the patient has a thick periodontal profile before surgery. A study did not find any correlations between BT and GT in healthy patients, but found an association between apicoincisal gingival width and the underlying BT [54]. Another study did not find statistically significant correlation between buccal bone thickness and soft tissue thickness—but its sample comprised patients with thin periodontal biotype (< 1 mm) [57]. The correlation between soft and hard tissue thickness remains inconclusive based on conflicting evidence. However, a meta-analysis demonstrated a significant association between a thick phenotype and a thicker alveolar plate [58]. The correlation results also indicate that the longer the AC-GM distance, the wider the interdental papilla will be at T_12_. Both are considered supracrestal soft tissues, and according to these results, they demonstrate a similar pattern of growth after ACL.

The ACL outcomes involve the adequate diagnosis of the causes related to the excessive gingival display and, consequently, adequate surgical planning with the selection of the most indicated technique. A rebound of 0.5 mm in the gingival margin is a substantial amount and should be taken into consideration in the treatment plan. Professional training is essential to offer the patient better outcomes, stability, and durability. For future research, it will be important to include the intra-oral scanner exam at T_0_, T_3_, T_6_, and T_12_ to assess the stability of the gingival margin, as it offers greater measurement reliability and, at the same time, does not present a high cost or expose the patient to radiation.

### Strengths and limitations

Some strengths of this study should be addressed compared with previous articles published on the same topic. A 12-month follow-up was performed to ensure a satisfactory stability analysis until the end of the healing process. The diagnostic methods included ST-CBCT, what provides a non-invasive analysis of periodontal phenotype and precise surgical plan. The correlation analysis is a novelty in comparison to previous studies, which could not specify how the clinical variables correlated to each other. Also, the dimensional change evaluation of interdental papilla is a lacking topic regarding ACL in APE patients. Furthermore, the absence of dropouts within the sample in this study enhances both statistical power and data integrity.

Some limitations should be pointed out in our study. Although in the present study the small sample is a limitation, several publications that deal with the same subject have samples of similar sizes, such as twenty one [46, 53]⁠, eighteen [42]⁠, fourteen [59],⁠ or six patients [50]⁠⁠. Furthermore, they have a follow-up of up to 6 months or there is no tomographic evaluation, among other factors [15, 16, 46]. Despite the sample size being similar to previous studies, it can still be considered small to take the results with clinical guidelines. Except for the CCL, no clinical measurements were taken during the immediate postoperative period, as well as tomographic measurements, although very well performed, can lead to some degree of distortion. Furthermore, the surgeries were performed by different students, which makes standardization difficult, even when supervised by the same specialists. The involvement of graduate students in conducting the surgical procedures introduces a potential bias on the validity of the results. The proficiency of these students could influence the outcomes, and depending on the context, the findings might not faithfully mirror the results achievable by experienced practitioners. The photographic analysis can be a limitation, considering that even the most suitable photographic protocol can provide some degree of distortion. It is important to acknowledge that inter-examiner analysis was not performed in this study, as the measurements were carried out exclusively by a single experienced examiner. However, our study stands out as one of the few studies about ACL stability that evaluated intra-examiner reliability through repeated measures with a 15-day interval, thoroughly examining both systematic and random errors. The clinical reproducibility of our findings might be limited since our sample was composed only of individuals with thick periodontal phenotypes. However, assessing gingival or periodontal phenotype can be performed through different methodologies: visual inspection, periodontal probe transparency in the gingival sulcus, transgingival direct measurements, trans-surgical measurements with calipers, and various imaging examinations such as ultrasound, radiographic exams, and ST-CBCT [15, 23, 53, 60]. The high variability in the definition of phenotypes and methods to assess them may lead to varied classifications of a patient’s condition [61]. Therefore, clinicians may find our results particularly relevant when treating patients with more than 1 mm gingival thickness, as measured specifically on ST-CBCT.

## Conclusions

The esthetic crown lengthening procedure has led to stability of the gingival margin at 3, 6, and 12 months postsurgically in individuals with shortened clinical crown length due to altered passive eruption. Although a certain degree of rebound was observed, this could be considered not clinically or statistically significant. During the healing period after esthetic crown lengthening, the probing depth had a tendency to restore its original length to some extent—mostly due to the migration of the gingival margin. The healing process leads to an increase in papilla height and a thickening in papilla width. The increase in papilla width resulted in clinical crowns appearing more triangular in shape than originally planned. This can be attributed to the changes in the soft tissue architecture during the healing process. The substantial increase in gingival thickness, bone thickness, distance between the alveolar crest, and the cementoenamel junction, as well as the distance between the alveolar crest and the gingival margin, implies that the esthetic crown lengthening procedure may lead to alterations in the periodontal phenotype. Strong positive correlations were found between clinical crown length and interdental papilla height at baseline, and interdental papilla width at 12 months and anatomical crown length. Other correlations, when significant, were moderate. Correlations can be used to manage changes in periodontal structures and to predict outcomes after the healing process. The findings emphasize the intricate nature of healing and adaptation in periodontal tissues after this procedure, underscoring the importance of understanding these dynamics for effective clinical management.

## Data Availability

All data supporting the findings of this study are available within the paper.
